# Mesenchymal stem cells therapy for acute kidney injury: A systematic review with meta-analysis based on rat model

**DOI:** 10.3389/fphar.2023.1099056

**Published:** 2023-04-13

**Authors:** Pingping Wanyan, Xin Wang, Nenglian Li, Yong Huang, Yali She, Li Zhang

**Affiliations:** ^1^ Department of Pathology and Pathophysiology, School of Basic Medicine, Gansu University of Traditional Chinese Medicine, Lanzhou, China; ^2^ Department of Orthopedics, The First Hospital of Lanzhou University, Lanzhou, China; ^3^ Department of Surgery, The First Clinical Medical College of Lanzhou University, Lanzhou, China

**Keywords:** mesenchymal stem cells, acute kidney injury, rat, systematic review, meta-analysis

## Abstract

**Objective**: To systematically evaluate the efficacy of mesenchymal stem cells (MSCs) for acute kidney injury (AKI) in preclinical studies and to explore the optimal transplantation strategy of MSCs by network meta-analysis with the aim of improving the efficacy of stem cell therapy.

**Methods**: Computer searches of PubMed, Web of Science, Cochrane, Embase, CNKI, Wanfang, VIP, and CBM databases were conducted until 17 August 2022. Literature screening, data extraction and quality evaluation were performed independently by two researchers.

**Results and Discussion**: A total of 50 randomized controlled animal studies were included. The results of traditional meta-analysis showed that MSCs could significantly improve the renal function and injured renal tissue of AKI rats in different subgroups. The results of network meta-analysis showed that although there was no significant difference in the therapeutic effect between different transplant routes and doses of MSCs, the results of surface under the cumulative ranking probability curve (SUCRA) showed that the therapeutic effect of intravenous transplantation of MSCs was better than that of arterial and intrarenal transplantation, and the therapeutic effect of high dose (>1×10^6^) was better than that of low dose (≤1×10^6^). However, the current preclinical studies have limitations in experimental design, measurement and reporting of results, and more high-quality studies, especially direct comparative evidence, are needed in the future to further confirm the best transplantation strategy of MSCs in AKI.

**Systematic Review Registration:** identifier https://CRD42022361199, https://www.crd.york.ac.uk/prospero.

## 1 Introduction

Acute kidney injury (AKI) is a syndrome in which renal function deteriorates rapidly within hours or days caused by various factors. The main pathological changes are decreased renal perfusion, renal tubular injury, tubulointerstitial inflammation and decreased glomerular filtration rate (Kellum et al., 2013). AKI is a global public health problem, affecting nearly 14 million patients and causing 1.7 million deaths each year (Mehta et al., 2015). In addition, 20% of inpatients were complicated with AKI, and half of them needed renal replacement therapy (Levey and James, 2017). Even mild and reversible AKI can have serious consequences such as death (Hoste et al., 2006; Uchino et al., 2006). The long-term consequences of AKI are the development and aggravation of chronic kidney disease and end-stage renal disease (Ishani et al., 2009; Chawla et al., 2014). Although the advances we have made in the pathogenesis of AKI, the available clinical treatment options are limited. Multiple treatment strategies, such as antioxidants, diuretics, dopamine, and reducing exposure to nephrotoxic drugs, do not change the course of the disease (Benoit and Devarajan, 2018). As a last resort for end-stage disease, renal replacement therapy has multiple complications and high fees, leaving patients with a great financial and emotional burden, and does not consistently enhance the recovery of kidney function (Caskey and Jager, 2014).

In recent years, advances in regenerative medicine have provided promising therapeutic strategies for the prevention or treatment of AKI. Among them, mesenchymal stem cells (MSCs) are mesoderm-derived stem cells with potential for self-renewal and multidirectional differentiation. Their wide range of sources, easy isolation, high migration capacity and expansion rate, as well as mild immune rejection and less ethical controversy make them a hotspot for research (Almalki and Agrawal, 2016; Peired et al., 2016). The renal protective effects of MSCs are mainly anti-inflammation, promoting angiogenesis, mobilization of endogenous stem cells, anti-apoptosis, anti-fibrosis, anti-oxidation, and promoting cell reprogramming (de Almeida et al., 2013). Studies have shown that bone marrow-derived stem cells can differentiate into a variety of inherent components of the kidney, including renal tubular epithelial cells, podocytes, mesangial cells, and capillary endothelial cells (Poulsom et al., 2001; Li et al., 2006). Based on the regenerative ability of MSCs and the tendency to move towards damaged tissue in many diseases, the therapeutic effect of MSCs has been explored through AKI animal models. Preclinical studies have shown that MSCs can secrete cytokines such as IL-6, IL-10, TGF- *β* and other cytokines against the early inflammatory environment of AKI (Bassi et al., 2012; Wang et al., 2012), differentiate into pericyte-like cells and promote angiogenesis and renal vascular perfusion by secreting vascular endothelial growth factor, insulin-like growth factor-1 and hepatocyte growth factor (Ball et al., 2007; Imberti et al., 2007; Au et al., 2008; Sanz et al., 2008). They can also promote the regeneration of injured renal cells by migrating to the kidney and differentiating into renal parenchyma cells (Humphreys and Bonventre, 2008).

Although MSCs has made great progress in the animal model of AKI, there are still some problems. Firstly, some studies have shown that MSCs not only fail to migrate to the site of kidney injury to perform repair due to the obstruction of organs such as lung and liver, but also activate more granulocytes to aggravate kidney injury due to immune response (Duffield and Bonventre, 2005; Duffield et al., 2005). Secondly, most studies have found that MSC can differentiate into renal parenchyma cells, including glomerular cells, glomerular mesangial cells and renal tubular epithelial cells under specific conditions. Therefore, it is believed that MSC can repair the kidney by differentiating into renal parenchyma cells (Yokoo et al., 2006; Togel et al., 2007) However, with the continuous understanding of MSCs, some studies have not observed the migration and differentiation of MSCs in the kidney (Bi et al., 2007; Hu et al., 2013). Thirdly, the clinical trial of MSCs therapy did not achieve the desired results. A multicenter, randomized, double-blind, placebo-controlled clinical trial of intra-arterial infusion of allogeneic MSCs in 156 patients with AKI after cardiac surgery showed that MSCs did not reduce the time for recovery of renal function, the use of dialysis or mortality (Swaminathan et al., 2018). Finally, the transplant route and dose of MSCs have been proved to be the key factors affecting its effectiveness, and then the current animal studies have not compared the different routes and doses of MSCs (Fazekas and Griffin, 2020; Shang et al., 2022).

Therefore, as the first study in the current field, we will comprehensively collect all the studies on the treatment of AKI with MSCs, systematically evaluate the therapeutic effect of MSCs in AKI, and explore the best transplantation strategy of MSCs through network meta-analysis, in order to provide reference for future animal experiments and clinical studies.

## 2 Materials and methods

This systematic review and meta-analysis followed the Preferred Reporting Items for Systematic Reviews and Meta-analyses guidelines and PICOs (Moher et al., 2009). The protocol for this study was registered on PROSPERO (CRD42022361199, https://www.crd.york.ac.uk/prospero).

### 2.1 Inclusion and exclusion criteria

#### 2.1.1 Patients & disease (P)

Rat models with AKI were included, and non-rat models such as rabbits, pigs, monkeys and dogs, or non-AKI animal models were excluded.

#### 2.1.2 Interventions (I)

Mesenchymal stem cells. Exclusion of stem cells that are modified or combined with other interventions such as erythropoietin, colony-stimulating factor, etc.

#### 2.1.3 Control (C)

Placebo controls or controls with different routes or doses between MSCs. Studies lacking a control group were excluded.

#### 2.1.4 Outcome (O)

Serum creatinine (SCr) and renal histology score.

#### 2.1.5 Type of study (S)

Randomised controlled studies.

#### 2.1.6 Exclusion criteria

Vitro experiments, clinical trials, reviews, opinion articles, conference abstracts, and non-published data were excluded.

### 2.2 Data sources and searches

Candidate studies were identified through searches of PubMed, Web of Science, Cochrane, Embase, CNKI, Wanfang, VIP, CBM databases from their inception until 17 August 2022. The following terms were combined to design the search strategy (acute kidney injury OR acute renal injury OR acute kidney failure OR acute renal failure OR acute renal insufficiency OR acute kidney insufficiency) AND (stem cell OR stem cells). Further details of the search strategy are shown in Supplementary Table S1. We also searched the reference lists of identified articles for further relevant papers.

### 2.3 Literature screening, data extraction, and risk of bias assessment

Two trained researchers selected the papers and stringently extracted the data based on the inclusion/exclusion criteria, and the selections were cross-checked. In the case of disagreement, a third researcher settled the conflict with a common consensus. Data were extracted according to the pre-established full-text data extraction checklist, including 1) Basic characteristics: authors, publication years, country, type of study, species, sex, body weight, age, sample size, modeling method, types, sources, timing, and routes of transplantation of MSCs, and control group. 2) Key elements of bias risk assessment. 3) Outcome measures: SCr and histology score. Risk of bias in animal studies was assessed using the internationally recognized SYRCLE risk of bias assessment tool (Hooijmans et al., 2014).

### 2.4 Statistical analysis

The mvmeta package of STATA 16.0 software was used to construct the network meta-analysis in the frequentist framework. Since the animal studies were exploratory, a random-effects model was used for statistical analysis, while standardized mean difference (SMD) was used as the effect analysis statistic and its 95% confidence interval (95% CI) was provided. *p* represents the significant level of the test, *p* < 0.05 means the difference is statistically significant. Correction-comparison funnel plots were used to evaluate the presence of bias due to small sample effects in the intervention network. A network plot was used to summarize the evidence network between different routes and doses of MSCs transplantation. The surface under the cumulative ranking probability curve (SUCRA) was used to determine the best transplantation method. We performed subgroup analyses by different transplantation route (arterial, venous, and intrarenal) and dose (high dose group >1×10^6^, low dose group ≤1×10^6^).

## 3 Results

**FIGURE 1:**
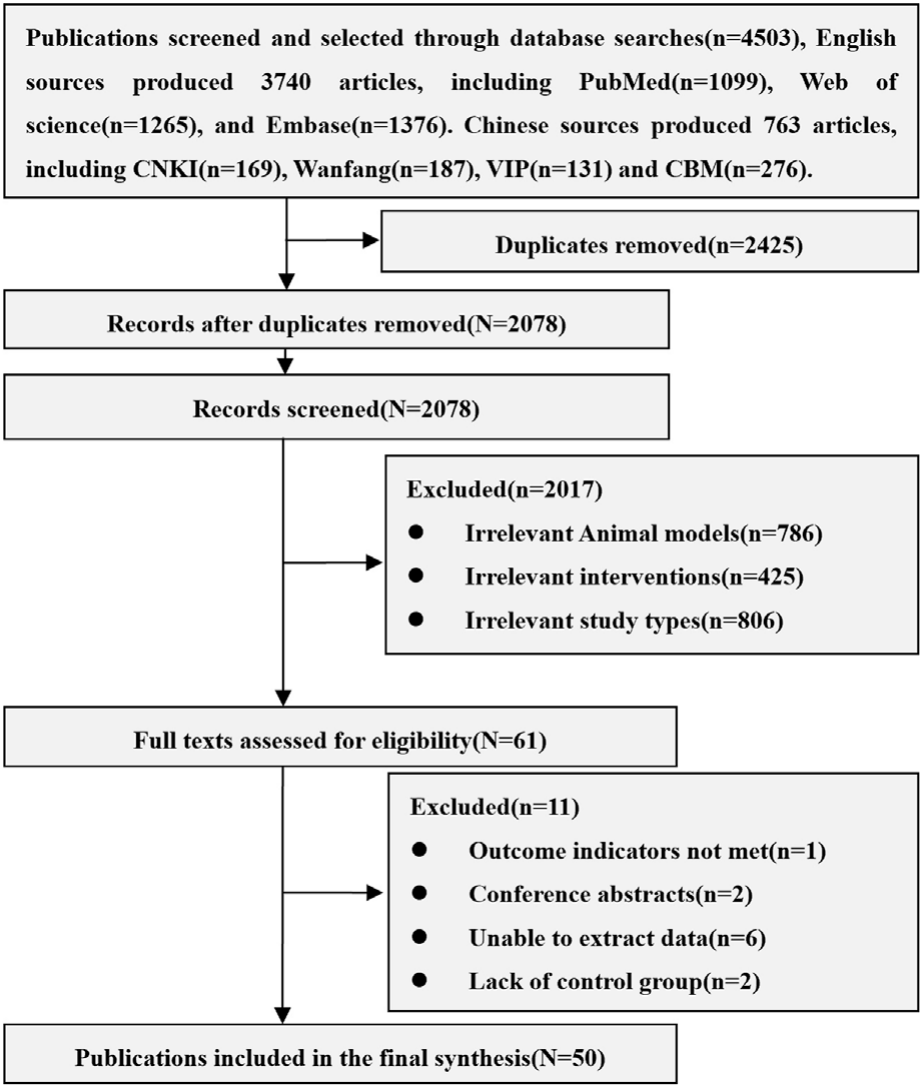
PRISMA flow chart with details of literature search.

A total of 4,503 related articles were obtained, including 3,740 in English and 763 in Chinese. After excluding repetitive, unrelated animal models (Human, mouse, dog and other AKI models, acute liver injury), unrelated interventions (Stem cell-derived exosomes and cytokines, modified stem cells, stem cells in combination with other treatments, non-stem cells) and unrelated research types (Reviews, conference abstracts, letters, case reports), 50 animal studies of MSCs in the treatment of AKI were included, including 38 English and 12 Chinese articles. The results of the literature search are shown in Figure 1.

### 3.1 Basic information of included studies

All the 50 studies included were randomized controlled studies. The varieties of rats include SD rats (40 studies), Wistar rat (7 studies), Lewis rat (2 studies) and Fisher 344 rat (1 study). The sex of rats is mainly male (45 studies). Rats weighed between 150 g and 370 g and were aged between 6 and 16 weeks, of which 4 studies did not report the weight of rats and 21 studies did not report the age of rats. The sample size is between 10 and 180. The models of AKI included clamping bilateral renal pedicles for 30–60 min (28 studies), clamping one renal pedicle for 45–60 min and resecting the other kidney (12 studies), and using lipopolysaccharide (1 study), glycerol (1 study), gentamicin (2 studies) and cisplatin (6 studies). The sources of MSCs include adipose tissue (21 studies), bone marrow tissue (18 studies), umbilical cord tissue (9 studies), fetal membrane tissue (2 studies), amniotic fluid (1 study) and endometrial lining (1 study). Isolation methods of MSCs include density gradient centrifugation (10 studies), adherent screening (16 studies), tissue digestion (14 studies), and flow cytometry separation (10 studies). The transplantation routes of MSCs include caudal vein (27 studies), penile vein (3 studies), femoral vein (2 studies), inferior vena cava (1 study), renal artery (6 studies), carotid artery (3 studies) and renal tissue (9 studies). The transplant dose of MSCs is between 0.5×10^6^ to 15×10^6^. The negative control group included Normal saline, PBS, Vehicle, Cultural medium, Blank, and DMEM. The basic information of included studies is shown in Supplementary Table S2.

### 3.2 Risk of bias assessment results

**FIGURE 2:**
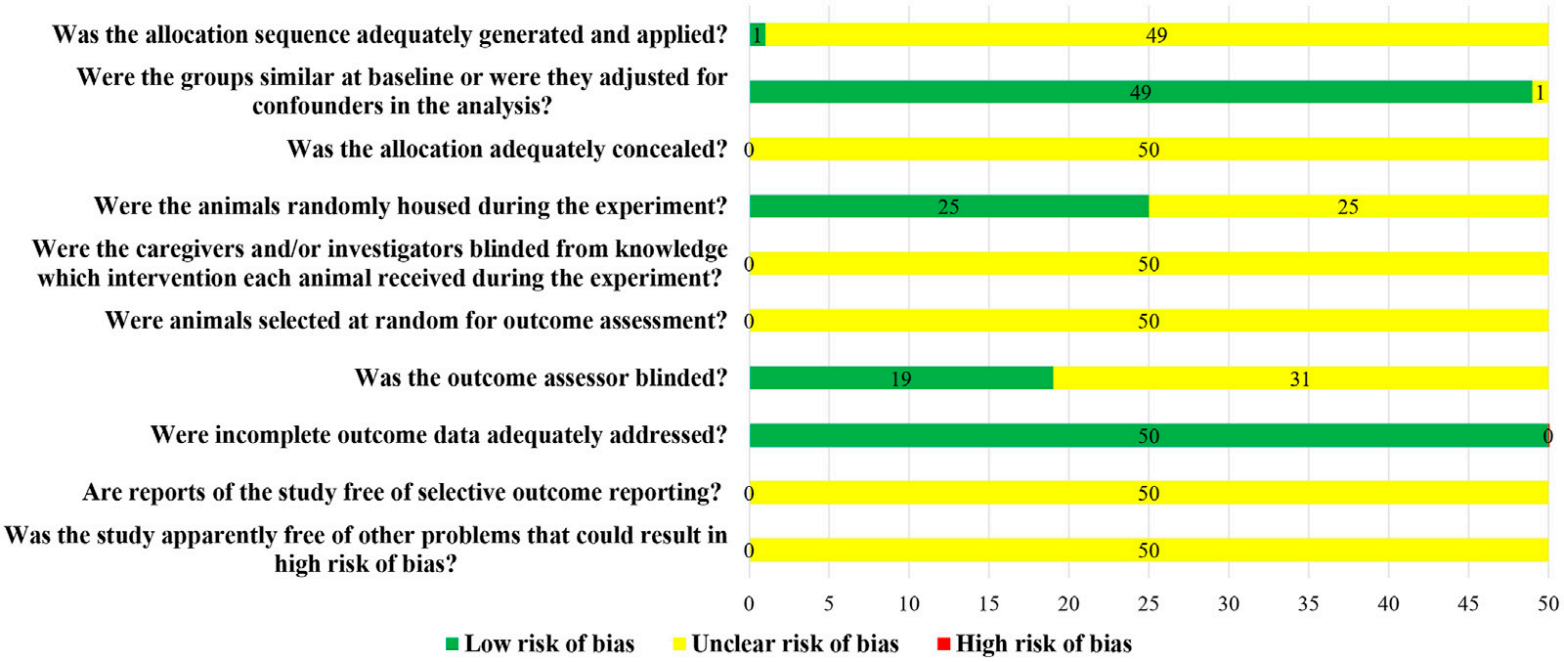
Risk of bias assessment of included studies.

Although all the 50 studies included were randomized controlled trials, only one study used a random number table to randomly group animals, and the other 49 studies did not report the method of random grouping. The baseline characteristics of animals, such as age, sex, and weight, were clearly reported in 49 studies. Twenty-five studies randomized animals during the experiment. None of the studies reported whether blind methods were applied to animal breeders and researchers, nor did they report whether animals were randomly selected for results measurement. Only 19 studies blinded the evaluators of the results. All the animals in the study were included in the result analysis, but no protocols were provided (Figure 2).

### 3.3 Meta-analysis results

#### 3.3.1 Comparison of different transplantation routes

##### 3.3.1.1 Results of traditional meta-analysis in high dose group

The results of traditional meta-analysis based on SCr and histological scores showed that the therapeutic effects of stem cells with different transplantation routes were significantly better than those in the placebo group. In addition, the therapeutic effect of stem cells *via* intravenous transplantation was higher than that of arterial and intrarenal transplantation (Table 1).

**TABLE 1:** Comparison of different transplantation routes in high dose groups.

Group	Meta-analysis	Number of studies	SMD	I^2^
SCr				
Vein vs. Placebo	Traditional	17	−6.79 [-8.51, −5.07]	90.5%
Intrarenal vs. Placebo	Traditional	6	−2.86 [-4.04, −1.68]	67.5%
Artery vs. Placebo	Traditional	4	−4.91 [-7.58, −2.23]	82.0%
Artery vs. Vein	Network	/	2.76 [-2.98, 8.50]	/
Artery vs. Intrarenal	Network	/	−1.78 [-8.26, 4.70]	/
Vein vs. Intrarenal	Network	/	−4.54 [-9.44, 0.36]	/
Histology score				
Vein vs. Placebo	Traditional	5	−9.54 [-14.44, −4.65]	93.2%
Intrarenal vs. Placebo	Traditional	6	−3.23 [-4.78, −1.68]	76.3%
Artery vs. Placebo	Traditional	3	−3.30 [-6.42, −0.19]	82.9%
Artery vs. Vein	Network	/	7.09 [-3.18, 17.36]	/
Artery vs. Intrarenal	Network	/	0.13 [-8.82, 9.08]	/
Vein vs. Intrarenal	Network	/	−6.96 [-15.82, 1.90]	/

##### 3.3.1.2 Results of network meta-analysis in high dose group

**FIGURE 3:**
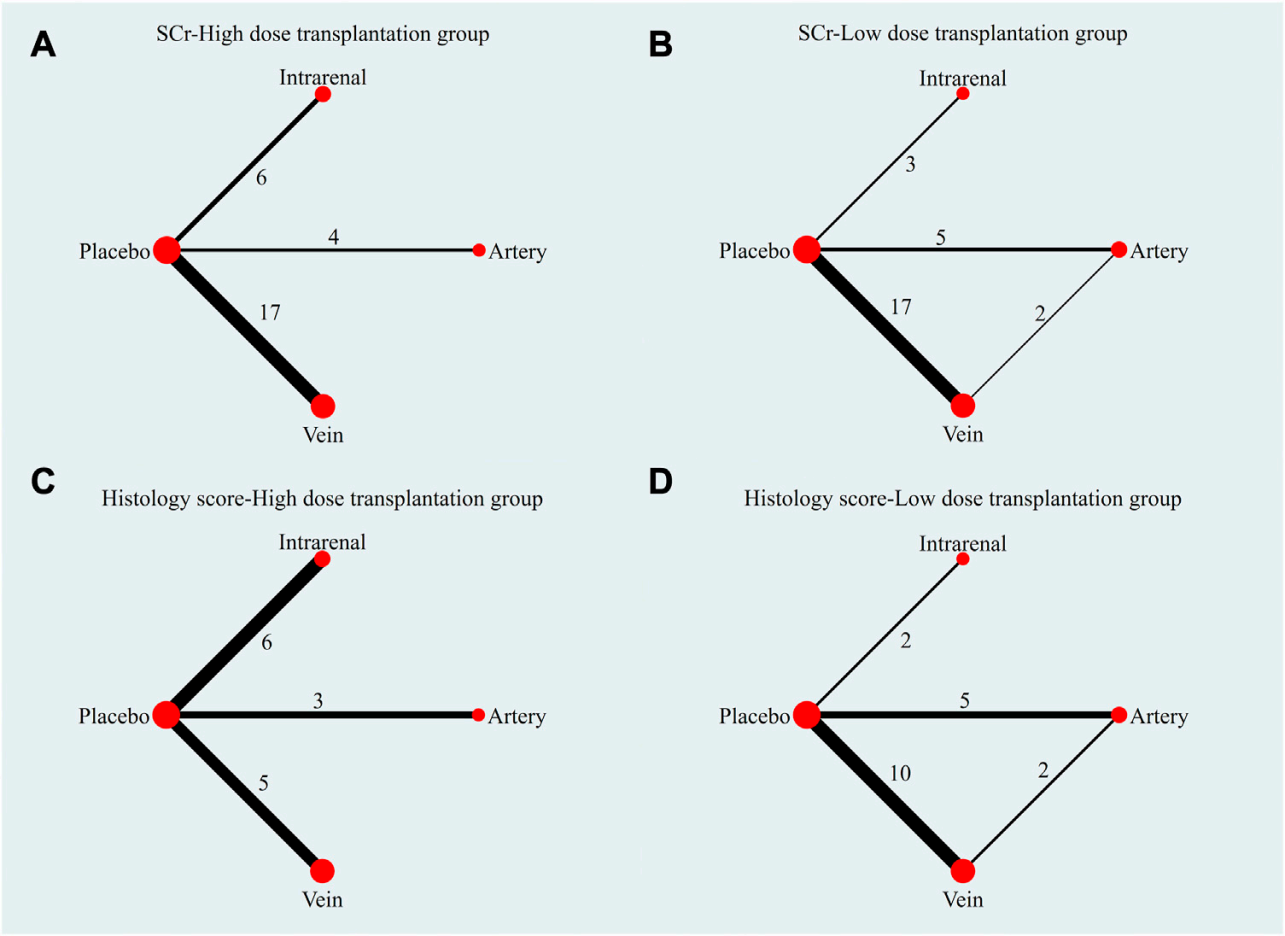
Evidence plot of different transplant routes. **(A)** SCr-High dose transplantation group. **(B)** SCr-Low dose transplantation group. **(C)** Histology score-High dose transplantation group. **(D)** Histology score-Low dose transplantation group.

**FIGURE 4:**
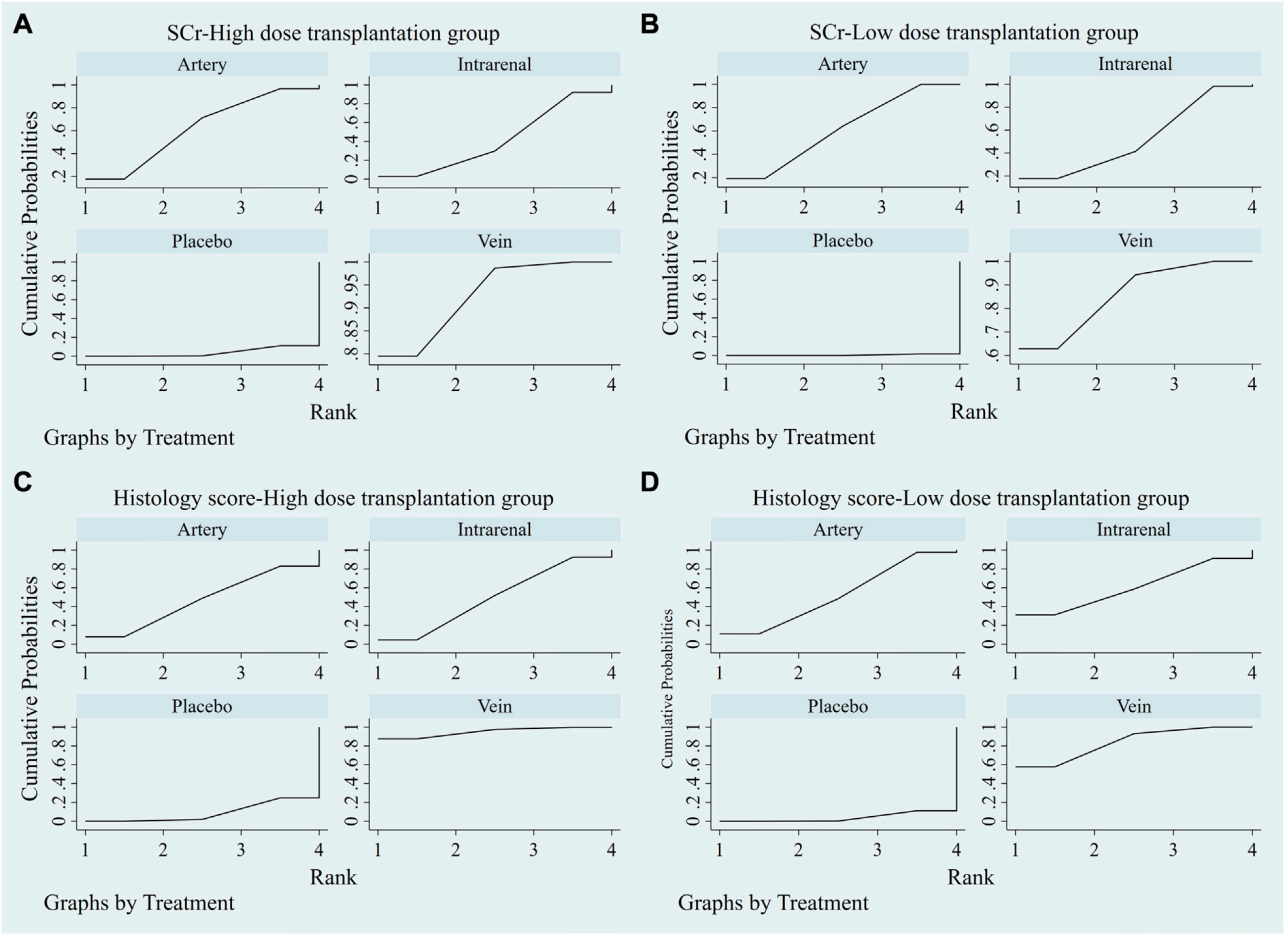
SUCRA plot of different transplantation routes. **(A)** SCr-High dose transplantation group. **(B)** SCr-Low dose transplantation group. **(C)** Histology score-High dose transplantation group. **(D)** Histology score-Low dose transplantation group.

**FIGURE 5:**
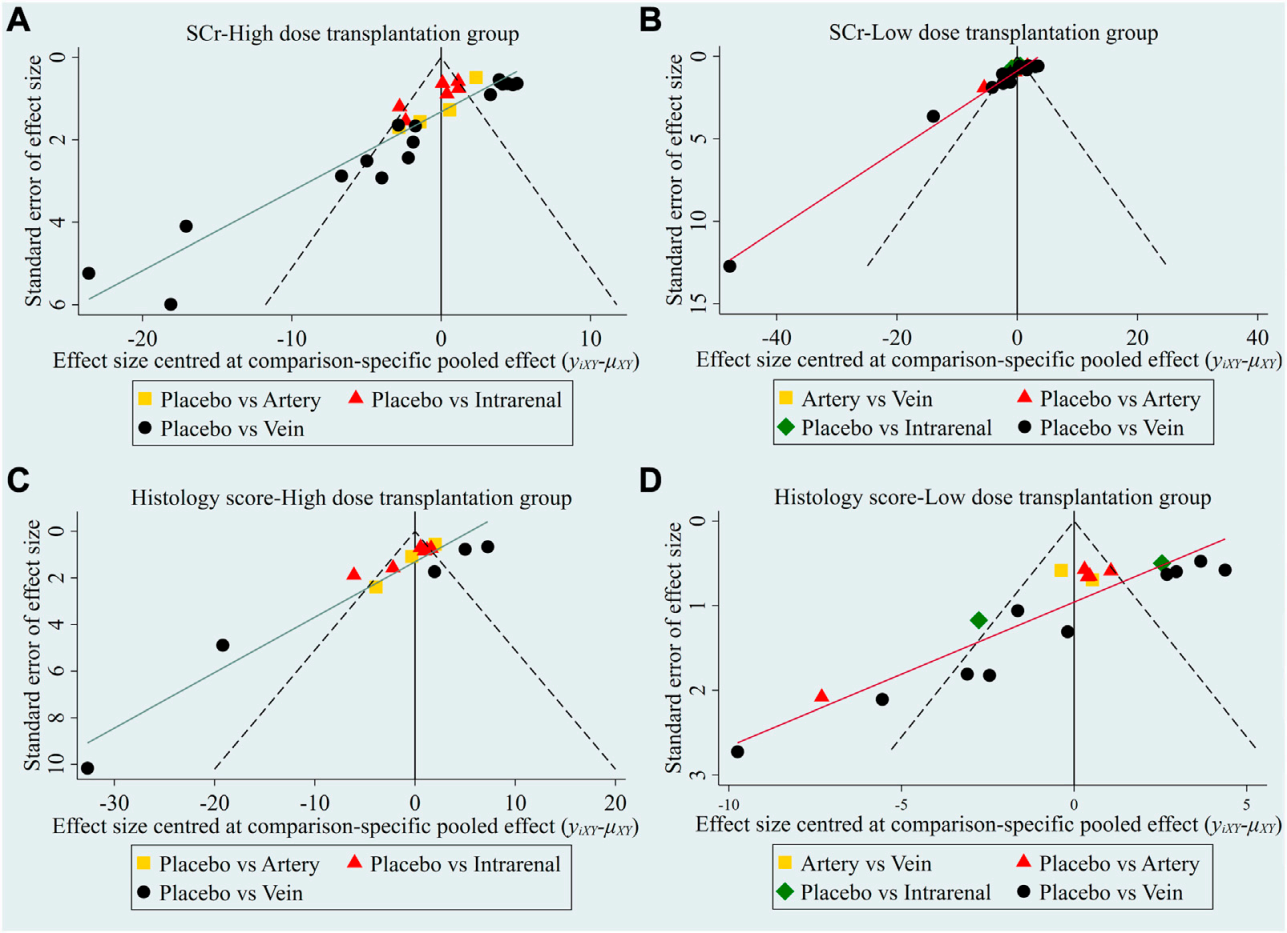
Comparison-correction funnel plot of different transplantation routes. **(A)** SCr-High dose transplantation group. **(B)** SCr-Low dose transplantation group. **(C)** Histology score-High dose transplantation group. **(D)** Histology score-Low dose transplantation group.

The evidence plot showed that there were no studies to compare different transplantation routes of stem cell (Figures 3A, C). The results of network meta-analysis based on SCr and histological scores showed that there was no significant difference in the therapeutic effect of stem cells with different transplantation routes (Table 1). The SUCRA results showed that the therapeutic effect of intravenous transplantation of stem cells was better than that of arterial and intrarenal transplantation (area under the curve: vein > artery > intrarenal > placebo) (Figures 4A, C). Asymmetric comparison-correction funnel plot suggests that there may be publication bias and small sample effect (Figures 5A, C).

##### 3.3.1.3 Results of traditional meta-analysis in low dose group

The results of traditional meta-analysis based on SCr and histological scores showed that the therapeutic effects of stem cells with different transplantation routes were significantly better than those in the placebo group. In addition, the therapeutic effect of stem cells *via* intravenous transplantation was higher than that of arterial and intrarenal transplantation (Table 2).

**TABLE 2:** Comparison of different transplantation routes in low dose groups.

Group	Meta-analysis	Number of studies	SMD	I^2^
SCr				
Vein vs. Placebo	Traditional	17	−4.17 [-5.42, −2.93]	87.8%
Intrarenal vs. Placebo	Traditional	3	−2.59 [-3.42, −1.76]	22.9%
Artery vs. Placebo	Traditional	5	−2.70 [-4.30, −1.09]	77.8%
Artery vs. Vein	Network	/	0.74 [-1.22, 2.70]	/
Artery vs. Intrarenal	Network	/	−0.42 [-3.43,2 .59]	/
Vein vs. Intrarenal	Network	/	−1.16 [-3.83, 1.51]	/
**Histology score**				
Vein vs. Placebo	Traditional	10	−4.89 [-6.75, −3.03]	90.7%
Intrarenal vs. Placebo	Traditional	2	−3.59 [-9.01, 1.83]	94.8%
Artery vs. Placebo	Traditional	5	−1.56 [-2.80, −0.32]	71.7%
Artery vs. Vein	Network	/	1.66 [-1.65, 4.98]	/
Artery vs. Intrarenal	Network	/	0.52 [-5.14, 6.19]	/
Vein vs. Intrarenal	Network	/	−1.14 [-6.48,4 .21]	/

##### 3.3.1.4 Results of network meta-analysis in low dose group

The evidence plot showed that two studies explored the therapeutic effects of different stem cell transplantation routes (Figures 3B, D). The results of network meta-analysis based on SCr and histological scores showed that there was no significant difference in the therapeutic effect of stem cells with different transplantation routes (Table 2). The SUCRA results showed that the therapeutic effect of intravenous transplantation of stem cells was better than that of arterial and intrarenal transplantation (area under the curve: vein > artery > intrarenal > placebo) (Figures 4B, D). Asymmetric comparison-correction funnel plot suggests that there may be publication bias and small sample effect (Figures 5B, D).

#### 3.3.2 Comparison of different transplant doses

##### 3.3.2.1 Results of traditional meta-analysis in artery transplantation group

The results of traditional meta-analysis based on SCr and histological scores showed that the therapeutic effects of different doses of stem cells were significantly better than those in the placebo group. In addition, the therapeutic effect of high-dose stem cells was higher than that of low-dose stem cells (Table 3).

**TABLE 3:** Comparison of different doses in artery transplantation group.

Group	Meta-analysis	Number of studies	SMD	I2
SCr				
High dose vs. Placebo	Traditional	4	−4.91 [-7.58, −2.23]	82.0%
Low dose vs. Placebo	Traditional	5	−2.70 [-4.30, −1.09]	77.8%
High dose vs. Low dose	Network	/	−1.83 [-4.64, 0.99]	/
Histology score				
High dose vs. Placebo	Traditional	3	−3.30 [-6.42, −0.19]	82.9%
Low dose vs. Placebo	Traditional	5	−1.56 [-2.80, −0.32]	71.7%
High dose vs. Low dose	Network	/	−0.66 [-2.68, 1.35]	/

##### 3.3.2.2 Results of network meta-analysis in artery transplantation group

**FIGURE 6:**
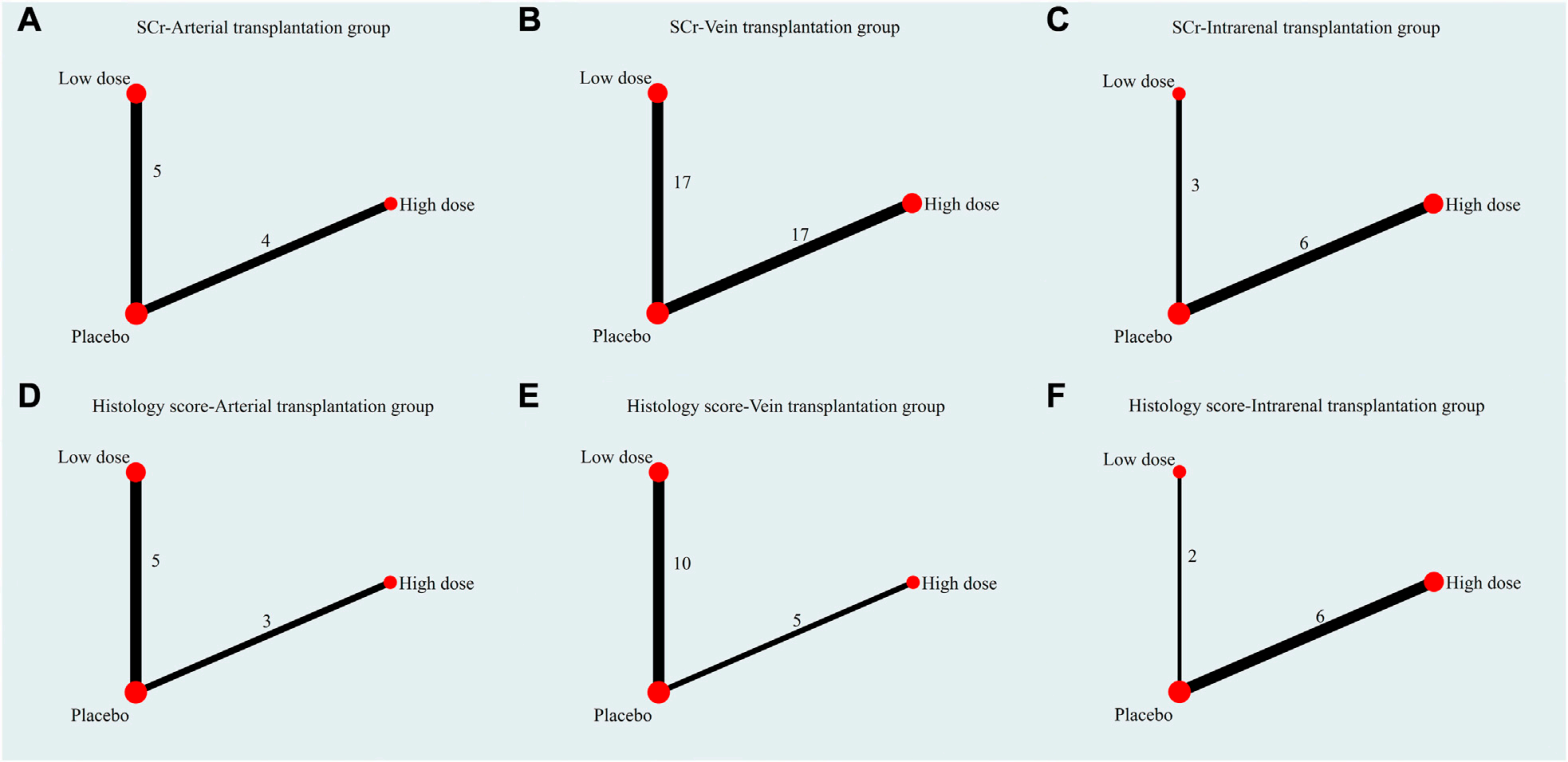
Evidence plot of different transplant doses. **(A)** SCr-Arterial transplantation group. **(B)** SCr-Vein transplantation group. **(C)** SCr-Intrarenal transplantation group. **(D)** Histology score-Arterial transplantation group. **(E)** Histology score-Vein transplantation group. **(F)** Histology score-Intrarenal transplantation group.

**FIGURE 7:**
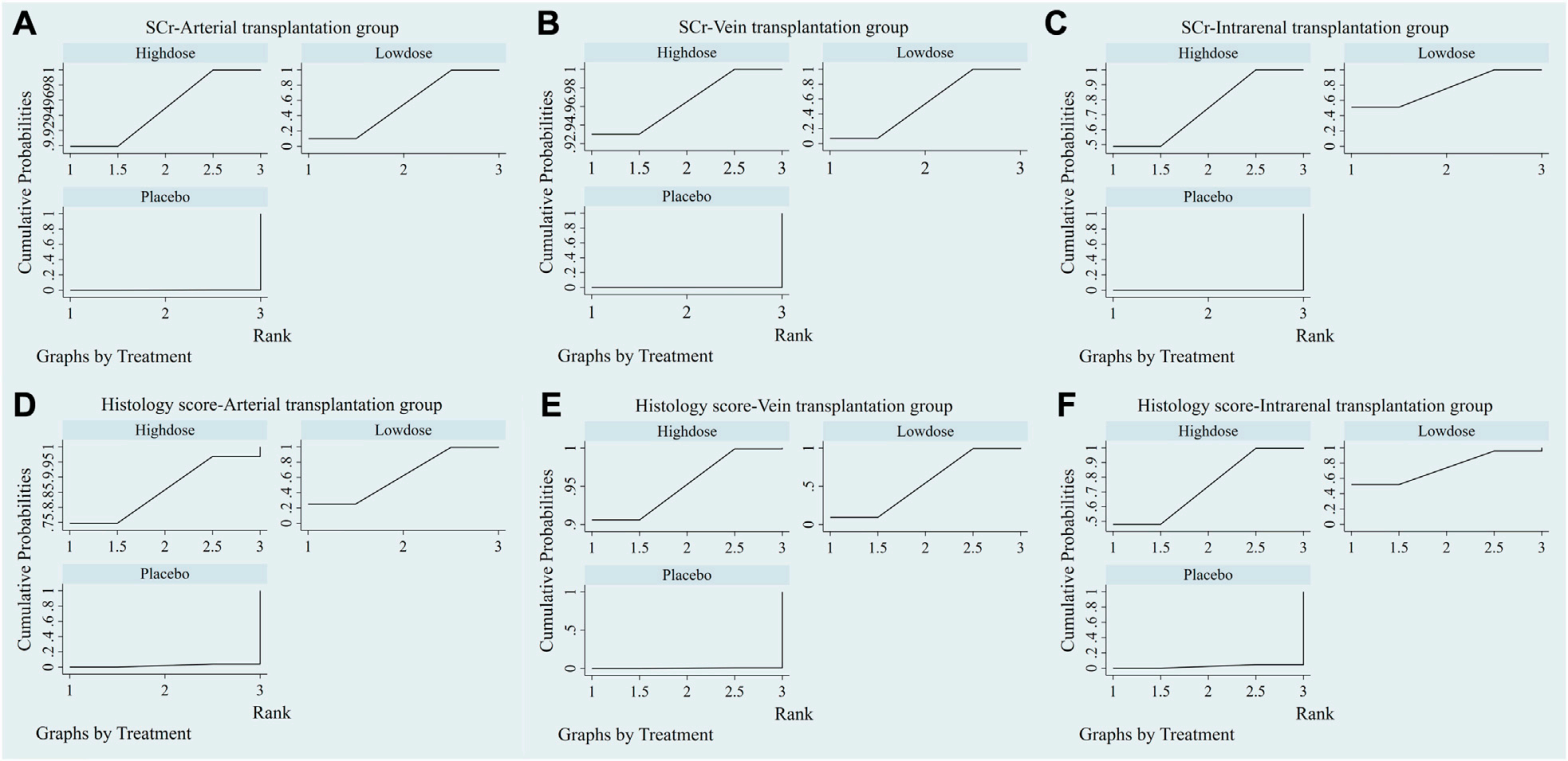
SUCRA plot of different transplantation doses. **(A)** SCr-Arterial transplantation group. **(B)** SCr-Vein transplantation group. **(C)** SCr-Intrarenal transplantation group. **(D)** Histology score-Arterial transplantation group. **(E)** Histology score-Vein transplantation group. **(F)** Histology score-Intrarenal transplantation group.

**FIGURE 8:**
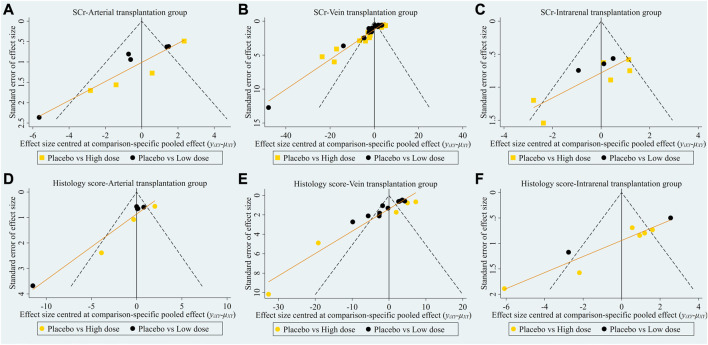
Comparison-correction funnel plot of different transplantation doses. **(A)** SCr-Arterial transplantation group. **(B)** SCr-Vein transplantation group. **(C)** SCr-Intrarenal transplantation group. **(D)** Histology score-Arterial transplantation group. **(E)** Histology score-Vein transplantation group. **(F)** Histology score-Intrarenal transplantation group.

The evidence plot showed that there is no study to compare the different transplantation doses of stem cell (Figures 6A, D). The results of network meta-analysis based on SCr and histological scores showed that there was no significant statistical difference between high-dose and low-dose stem cells (Table 3). SUCRA results showed that the therapeutic effect of high-dose stem cells was better than that of low-dose stem cells (area under the curve: high-dose group > low-dose group > placebo) (Figures 7A, D). Asymmetric comparison-correction funnel plot suggests that there may be publication bias and small sample effect (Figures 8A, D).

##### 3.3.2.3 Results of traditional meta-analysis in vein transplantation group

The results of traditional meta-analysis based on SCr and histological scores showed that the therapeutic effects of different doses of stem cells were significantly better than those in the placebo group. In addition, the therapeutic effect of high-dose stem cells was higher than that of low-dose stem cells (Table 4).

**TABLE 4:** Comparison of different doses in vein transplantation group.

Group	Meta-analysis	Number of studies	SMD	I^2^
SCr				
High dose vs. Placebo	Traditional	17	−6.79 [-8.51, −5.07]	90.5%
Low dose vs. Placebo	Traditional	17	−4.17 [-5.42, −2.93]	87.8%
High dose vs. Low dose	Network	/	−2.78 [-6.47, 0.91]	/
**Histology score**				
High dose vs. Placebo	Traditional	5	−9.54 [-14.44, −4.65]	93.2%
Low dose vs. Placebo	Traditional	10	−4.89 [-6.75, −3.03]	90.7%
High dose vs. Low dose	Network	/	−5.51 [-13.82, 2.81]	/

##### 3.3.2.4 Results of network meta-analysis in vein transplantation group

The evidence plot showed that there is no study to compare the different transplantation doses of stem cell (Figures 6B, E). The results of network meta-analysis based on SCr and histological scores showed that there was no significant statistical difference between high-dose and low-dose stem cells (Table 4). SUCRA results showed that the therapeutic effect of high-dose stem cells was better than that of low-dose stem cells (area under the curve: high-dose group > low-dose group > placebo) (Figures 7B, E). Asymmetric comparison-correction funnel plot suggests that there may be publication bias and small sample effect (Figures 8B, E).

##### 3.3.2.5 Results of traditional meta-analysis in intrarenal transplantation group

The results of traditional meta-analysis based on SCr and histological scores showed that the therapeutic effects of different doses of stem cells were significantly better than those in the placebo group. In addition, the therapeutic effect of high-dose stem cells was higher than that of low-dose stem cells (Table 5).

**TABLE 5:** Comparison of different doses in intrarenal transplantation group.

Group	Meta-analysis	Number of studies	SMD	I^2^
SCr				
High dose vs. Placebo	Traditional	6	−2.86 [-4.04, −1.68]	67.5%
Low dose vs. Placebo	Traditional	3	−2.59 [-3.42, −1.76]	22.9%
High dose vs. Low dose	Network	/	−0.01 [-1.57,1.55]	/
Histology score				
High dose vs. Placebo	Traditional	6	−3.23 [-4.78, −1.68]	76.3%
Low dose vs. Placebo	Traditional	2	−3.19 [-9.01, 1.83]	94.8%
High dose vs. Low dose	Network	/	0.09 [-4.34, 4.52]	/

##### 3.3.2.6 Results of network meta-analysis in intrarenal transplantation group

The evidence plot showed that there is no study to compare the different transplantation doses of stem cell (Figures 6C,F). The results of network meta-analysis based on SCr and histological scores showed that there was no significant statistical difference between high-dose and low-dose stem cells (Table 5). SUCRA results showed that the therapeutic effect of low-dose stem cells was better than that of high-dose stem cells (area under the curve: low-dose group > high-dose group > placebo) (Figures 7C, F). Asymmetric comparison-correction funnel plot suggests that there may be publication bias and small sample effect (Figures 8C, F).

#### 3.3.3 Sensitivity analysis

**FIGURE 9:**
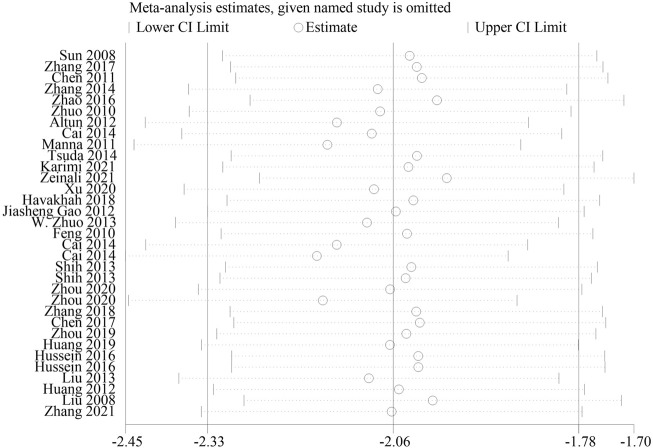
Sensitivity analysis results.

As histological score is a relatively subjective outcome index, in order to explore whether it will affect the reliability of meta-analysis results, we conducted a sensitivity analysis. The result showed that after one by one exclusion of certain studies, directions of the confidence intervals of the combined results of the remaining studies did not change, indicating that findings from the meta-analysis were robust and reliable (Figure 9).

## 4 Discussion

### 4.1 Summary of evidence

As a common emergency in internal medicine, AKI has a high incidence and relatively limited treatment, so it is in urgent need of new treatment. Cell therapy is considered to be promising. MSCs-based therapy is more and more widely used in clinic, especially in model animals, the basic research of MSCs has gradually laid a solid foundation for its clinical application. Many issues remain to be addressed before MSCs for AKI can enter clinical practice, including rigorous confirmation of the efficacy of MSCs and exploration of the optimal transplantation route and the number of cells required. Although the results obtained from animal models may not be fully applicable to the clinic, basic research is very important for stem cell therapy. Inflammatory cells such as macrophages, neutrophils and T cells can promote the development of AKI (Klausner et al., 1989; Rabb et al., 1996). MSCs has a strong immunomodulatory effect on these immune cells through intercellular contact and secretion of cytokines, chemokines and growth factors (Meirelles Lda et al., 2009; Bassi et al., 2012). Our traditional meta-analysis showed that whether it was based on arterial, intravenous, or intrarenal transplantation of MSCs, or high-dose or low-dose MSCs, the effect was significantly better than that of the placebo group. This fully proves the therapeutic potential of MSCs. On this basis, it is urgent to further explore the best transplantation strategy of MSCs in order to improve its therapeutic effect, especially when MSCs therapy has been tried in clinic. Under the guidance of the best transplantation strategy, patients can get the greatest benefit.

The route to transplant MSCs is the key factor to determine its fate (Togel et al., 2008). Our evidence network shows that the transplantation of MSCs includes venous, arterial and intrarenal approaches. The location and persistence of MSCs transplanted in different routes *in vivo* is controversial. The most common route is vein transplantation, because it is relatively safe and convenient. However, Cheng et al. found that 1 hour after intravenous transplantation of MSCs, most of the radiolabeled MSCs was concentrated in the lung (62%), followed by liver (12.5%), spleen (11.4%) and kidney (5.4%). And 1 week later, MSCs was not found in any organ (Cheng et al., 2013). Eggenhofer et al. also found that the MSCs of vein transplantation was initially concentrated in the lung and liver, and then gradually disappeared (Eggenhofer et al., 2012). On the contrary, studies have shown that intravenous MSCs can be directly homed to the damaged kidney or mobilized from the lung to the damaged kidney, thus significantly improving renal function (Wise et al., 2014). In addition, some studies believe that the main role of exogenous MSCs is to enhance the endogenous stem cells of the kidney to play a role (Humphreys and Bonventre, 2008; Humphreys et al., 2008). Injecting MSCs directly into the damaged kidney will ensure that more cells are delivered to the kidney. Even if this is not the best way, it will reduce the amount of MSCs that needs to be injected. Although it is difficult to clarify the best way of transplantation of MSCs from the mechanism level, in order to resolve this dispute, it is also important to explore the best way of transplantation of MSCs from the aspects of renal function and histological score. The results of network meta-analysis showed that although there was no significant difference in the repair effect among the three transplantation routes, the SUCRA plot provided valuable information, suggesting that the effect of intravenous transplantation of MSCs was better than that of arterial and intrarenal transplantation. The possible reason is that although intrarenal transplantation can effectively transport MSCs locally, the cells tend to gather at the injection site rather than spread to the kidney (Mias et al., 2008). Intrarenal transplantation may also aggravate the secondary damage of the kidney and adversely affect the recovery of renal function (Moustafa et al., 2016). In addition, studies have shown that although arterial transplantation can significantly reduce the capture of MSCs in the lungs and increase the chances of homing to the kidney. However, MSCs is widely distributed in other organs, not kidneys (Li et al., 2009; Cai et al., 2014). Compared with vein transplantation, arterial puncture is highly invasive in AKI rat model, resulting in massive bleeding and more serious secondary injury (Monteiro Carvalho Mori da Cunha et al., 2013). The prevailing view is that although vein transplantation cannot be distributed to the kidney in large quantities, they secrete various cytokines such as VEGF, HGF, IGF-1 and SDF-1 through paracrine or endocrine pathways, exerting immunomodulatory and anti-inflammatory effects, or significantly ameliorating kidney injury in AKI rats by releasing microvesicles containing various bioactive factors (Burst et al., 2010; Bianchi et al., 2014; Luo et al., 2014; Luk et al., 2016). Therefore, intravenous transplantation of stem cells for AKI may be a promising option for the future. However, we should also understand that the large number of dead and/or dying MSCs retained in the pulmonary or hepatic circulation after intravenous infusion may place some burden on the diseased organism.

The transplantation dose of MSCs is the key factor to determine its therapeutic effect. Although there are no clinical results on MSCs in the treatment of AKI, transplantation doses of MSCs vary from 0.3×10^6^ to 3×10^6^ in a clinical trial of patients with septic shock. Although there was no pre-specified signal of MSCs-related or severe adverse events and disease improvement during follow-up, an improvement in plasma cytokine profile was observed in the cohort of the highest dose of MSCs, suggesting the effect of transplant dose on the therapeutic efficacy of MSCs (McIntyre et al., 2018; Schlosser et al., 2019). Therefore, further research is needed to explore the optimal transplantation dose of MSCs in order to obtain the best clinical effect. Some studies have shown that 1×10^5^ of MSCs is more effective than 1×10^6^ of MSCs transplanted through carotid artery or tail vein in improving renal injury and function of AKI. Because they found that 1×10^6^ of stem cells increased the risk of vascular embolism, resulting in a significant reduction in blood flow signals to the kidneys (Cai et al., 2014). It has also been suggested that most of the regenerated cells in the kidney are derived from renal cells, and the role of MSCs is to enhance the proliferation of these cells (Humphreys and Bonventre, 2008; Humphreys et al., 2008). Therefore, the degree of renal regeneration after AKI may be limited by the regenerative potential of its endogenous cells, but has nothing to do with the number of transplanted MSCs. On the contrary, some studies have suggested that the transplantation dose of MSCs should be between 1 and 3×10^6^ in order to meet the dose needed for intravascular transplantation (Kunter et al., 2006; De Martino et al., 2010; Zhuo et al., 2013). The transplantation dose of MSCs included in the study ranged from 0.5×10^6^ to 15×10^6^. In order to obtain valuable information, we regard more than 1 × 10^6^ as a high-dose group and *vice versa* as a low-dose group. Although the results of network meta-analysis showed that there was no significant difference in the therapeutic effect of different doses of MSCs, the results of the subgroup analyzed by traditional meta-analysis showed that the therapeutic effect of high dose of MSCs was higher than that of low dose of MSCs, and the SUCRA plot also suggested that high dose of MSCs had greater therapeutic potential. It is worth noting that traditional meta-analysis showed that the therapeutic effect of high-dose MSCs was better than that of low-dose MSCs, but the results of network meta-analysis showed that there was no significant difference between the two groups. In fact, the traditional meta-analysis only judged the therapeutic effect of high-dose and low-dose MSCs from the size of the effect, and did not directly compare the relationship between them, nor could it get the results of statistical difference. In contrast, network meta-analysis showed a statistical correlation between high-dose and low-dose group through indirect comparison. Therefore, the results of network meta-analysis are more accurate. In conclusion, we should be aware of the fact that too low dose of MSCs is difficult to produce sufficient therapeutic effect, but too high dose is not a good thing. The results of Shani et al. showed that there is a curved relationship between the improvement of renal function and the transplant dose of MSCs. When the transplantation dose of MSCs increased from 1.5×10^5^ to 1×10^6^, the change of renal function became worse gradually, and then improved gradually (Zilberman-Itskovich et al., 2019). But they did not further increase the transplantation dose of MSCs. Therefore, it is necessary to further explore the best transplantation dose of MSCs at the level of 1×10^6^ and above in the future.

### 4.2 Evidence quality

The inherent bias risk of each study was assessed by SYRCLE’s risk of bias tool to explore the evidence quality. We find that there is a certain risk of bias of current studies.

One) Selection bias: Random grouping and covert grouping of animals while ensuring the balance of their baseline characteristics is an important measure to reduce selective bias (Hooijmans et al., 2014). Although all 50 studies were randomized controlled trials and the baseline characteristics of animals were balanced, 49 studies did not report randomized grouping methods and the implementation of covert grouping, resulting in a large selective bias. 2) Implementation bias: The placement of animals, such as different light and temperature, will affect the experimental results (Hooijmans et al., 2014; Park et al., 2018). However, only half of the studies randomized animals. In addition, no research has blinded animal breeders and researchers, resulting in a certain implementation bias. 3) Measurement bias: Failure to apply blind method to evaluators in animal studies may lead to exaggeration of effect and produce false positive results (Sena et al., 2007; Vesterinen et al., 2010). However, only 19 studies applied blind methods to the evaluators of the results, but none of them reported the specific process of blindness. Therefore, future research should pay more attention to the application of blind method in experimental design, and more experimental details should be provided to improve the reporting quality of animal studies. 4) Report bias: Selectively reporting the results of the study will lead to publication bias and affect the reliability of the experimental results (Korevaar et al., 2011). The protocol is not available for all studies, and it is not certain whether they report all the results unselectively in accordance with the prior plan. 5) Publication bias: Negative animal results are difficult to publish (Ioannidis, 2012). Systematic reviews that do not include negative findings will overestimate the effect of interventions. We can conclude that there may be some publication bias in this field by asymmetric comparison-correction funnel plots.

### 4.3 Strengths and limitations of this study

Strengths: 1) This is the first study in the current field to explore the optimal transplantation strategy of MSCs by systematic review and network meta-analysis. 2) We only explored the effect of MSCs on AKI in rats, therefore, there is less heterogeneity between studies and the results are more accurate and targeted. 3) We explored the best transplantation route and dose of MSCs in different subgroups, and the results of different subgroups were consistent, which fully proved the reliability of results. 4) Although MSCs from different sources have different characteristics, which are mainly reflected in the differences of proliferation rate, cytokine profiles and immunomodulatory ability, an important feature of MSCs from different sources is that they have low expression of major histocompatibility complex (MHC) class I molecules and do not express MHC class II molecules, which means that they can be transplanted across MHC barrier without causing obvious immune rejection (Miceli et al., 2021). In addition, the basic characteristics of the surface markers of MSCs are that they express cluster of differentiation CD105, CD73 and CD90, but do not express CD45, CD34, CD14 or CD11b, CD79a or CD19. This shows that MSCs from different sources have high homogeneity, and more data will be obtained when combined for analysis, thus providing stronger evidence. Therefore, we combine MSCs from different sources to analyze them.

Limitations: 1) Because the transplantation dose of MSCs varies greatly among different studies, we can only divide it into high-dose group and low-dose group, so it is difficult to make further dose division. 2) The therapeutic effects of MSCs in different strains of rats may be different, but due to the limitation of the number of studies, we did not consider the influence of rat strains on the results of meta-analysis. 3) The comparison-correction funnel plot indicates that there may be publication bias, which affects the accuracy of the results. We did not search the gray literature and conference abstracts, which also increased the possibility of publication bias.

## 5 Conclusion

To determine the therapeutic effect of MSCs and the best transplantation strategy is very important for the treatment of AKI. The results of traditional meta-analysis and network meta-analysis based on 50 studies showed that MSCs could significantly improve the renal function and injured renal tissue of AKI rats, and the effect of intravenous transplantation of MSCs was better than that of arterial and intrarenal transplantation, and the effect of high dose transplantation was better than that of low dose transplantation. However, limitations of the current study in terms of experimental design, outcome measurement and reporting reduce the reliability of the conclusions. More high-quality animal experiments, especially evidence of direct comparisons, are needed in the future to explore the best transplantation strategy for MSCs.

## Data Availability

The original contributions presented in the study are included in the article/Supplementary Material, further inquiries can be directed to the corresponding author.
