# Supplementary material for: Mesenchymal stem cells therapy for acute kidney injury: A systematic review with meta-analysis based on rat model
Source: Front Pharmacol. 2023 Apr 13;14:1099056. doi: 10.3389/fphar.2023.1099056 (PMC10133560; doi:10.3389/fphar.2023.1099056)
Supplement: Supplementary file 1 [file Table1.DOCX]

**Mesenchymal Stem Cells Therapy for Acute Kidney Injury: A Systematic Review with Meta-analysis Based on Rat Model**

**Table 1: Chinese and English search strategies**

| Comments:  In order for non-Chinese readers to understand the Chinese search strategy of this article, we translated the Chinese search terms in the search formula.  **PubMed**  #1: "stem cell"[Title/Abstract] OR "stem cells"[Title/Abstract] 321,220  #2: "Stem Cells"[MeSH Terms] 251,984  #3: #1 OR #2 413,965  #4: "acute kidney injury"[Title/Abstract] OR "acute renal injury"[Title/Abstract] OR "acute kidney failure"[Title/Abstract] OR "acute renal failure"[Title/Abstract] OR "acute renal insufficiency"[Title/Abstract] OR "acute kidney insufficiency"[Title/Abstract] 60,622  #5: "Acute Kidney Injury"[MeSH Major Topic] 42,820  #6: #4 OR #5 71,744  #7: #3 AND #6 1,099  **WOS**  (TS=(stem cell OR stem cells)) AND TS=(acute kidney injury OR acute renal injury OR acute kidney failure OR acute renal failure OR acute renal insufficiency OR acute kidney insufficiency) 1265  **Embase**  #1: 'stem cell':ab,ti OR 'stem cells':ab,ti 462, 292  #2: 'stem cells'/exp 444,197  #3: #1 OR #2 607, 091  #4: 'acute kidney injury':ab,ti OR 'acute renal injury':ab,ti OR 'acute kidney failure':ab,ti OR 'acute renal failure':ab,ti OR 'acute renal insufficiency':ab,ti OR 'acute kidney insufficiency':ab,ti 90, 996  #5: 'acute kidney injury'/exp 115, 600  #6: 'acute renal injury'/exp 46, 231  #7: #4 OR #5 OR #6 171, 851  #8: #3 AND #7 ('article'/it OR 'review'/it) 1376  **CNKI/高级检索; CNKI / Advanced Search (文献来源:SCI来源期刊 EI来源期刊 核心期刊 CSSCI CSCD)**  主题:急性肾损伤 AND (干细胞 OR 万能细胞) 169  Subject: acute kidney injury AND (stem cell OR stem cells) 169  **万方/高级检索; Wanfang database/ Advanced Search**  主题: 急性肾损伤AND (干细胞 OR 万能细胞) 187  Subject: acute kidney injury AND (stem cell OR stem cells) 187  **VIP/高级检索; VIP database/ Advanced Search**  题名或关键词: 急性肾损伤 AND (干细胞 OR 万能细胞) 131  Title or keyword: acute kidney injury AND (stem cell OR stem cells) 131  **CBM/高级检索; CBM / Advanced Search**  #1: "急性肾损伤"[不加权:扩展] 18427  #2: "急性肾损伤"[常用字段:智能] OR "急性肾衰竭"[常用字段:智能] OR "急性肾功能不全"[常用字段:智能] 88091  #3: #1 OR #2 88091  #4: "干细胞"[不加权:扩展] 378585  #5: "干细胞"[常用字段:智能] 705257  #6: #4 OR #5 705257  #7: #3 AND #6 276  #1:" acute kidney injury "[unweighted, extended] 18427  #2:" acute kidney injury "[ common field: smart] OR "acute renal failure" [ common field: smart] OR "acute renal insufficiency" [ common field: smart] 88091  #3: #2 OR #3 88091  #4:"Stem cell"[unweighted, extended] 378585  #5:" Stem cell "[ common field: smart] OR " stem cells"[ common field: smart] 705257  #6: #4 OR #5 705257  #7: #3 AND #6 276 |
| --- |

**Table 2: Basic information of included studies**

| Author  (Year) | Country | Type of study | Species | Sex | Body weight | Age | Sample size (E/C) | Modeling method | Stem cells transplantation | | | | Isolation/validation procedure of stem cells | Control |
| --- | --- | --- | --- | --- | --- | --- | --- | --- | --- | --- | --- | --- | --- | --- |
|  |  |  |  |  |  |  |  |  | Sort | Source | Route | Dose |  |  |
| Sun 2008[1] | China | RCT | SD rat | Male | 200-300g | 7 weeks | 10/20 | Glycerol | MSCs | Bone marrow of SD rats | Renal artery | 2×10^6^ | Density gradient centrifugation | PBS |
| Xiu 2018[2] | China | RCT | SD rat | Female | 180-220g | / | 15/15 | Lipopolysaccharide | MSCs | Bone marrow of SD rats | Tail vein | 1×10^6^ | Adherent screening | Blank |
| Zhang 2017[3] | China | RCT | SD rat | Male | 350-370g | 11-12 weeks | 6/6 | Clip bilateral renal pedicles for 40 minutes | MSCs | Adipose tissue in the groin of SD rats | Tail vein | 2×10^6^ | Tissue digestion | PBS |
| Chen 2011[4] | China | RCT | SD rat | Male | 275-300g | Adult | 8/8 | Clip bilateral renal pedicles for 60 minutes | MSCs | Adipose tissue around epididymis in SD rats | Intrarenal | 3×10^6^ | Tissue digestion | Cultural medium |
| Zhang 2014[5] | China | RCT | SD rat | Male | / | Adult | 25/25 | Clip bilateral renal pedicles for 40 minutes | MSCs | Human adipose tissue | Renal cortex | 2×10^6^ | Tissue digestion | Normal saline |
| Zhao 2016[6] | China | RCT | SD rat | Male | 200-250g | / | 10/10 | The right renal pedicle was clamped for 50 minutes, and the left kidney was removed. | MSCs | Adipose tissue around epididymis in SD rats | Renal cortex | 1×10^6^ | Tissue digestion | PBS |
| Hafazeh 2019[7] | China | RCT | Wistar rat | Male | 180-200g | Adult | 7/7 | Clip bilateral renal pedicles for 45 minutes | MSCs | Adipose tissue in the groin of SD rats | Tail vein | 2×10^6^ | Flow cytometry separation | PBS |
| Erpicum 2017[8] | China | RCT | Lewis rat | Male | / | 8-10 weeks | 9/6 | The right renal pedicle was clamped for 45 minutes, and the left kidney was removed. | MSCs | Bone marrow of Lewis rats | Tail vein | 1.5×10^6^ | Density gradient centrifugation | Normal saline |
| Zhuo 2010[9] | China | RCT | SD rat | Male | 250-300g | 8-10 weeks | 8/8 | The right renal pedicle was clamped for 60 minutes, and the left kidney was removed | MSCs | Bone marrow of SD rats | Tail vein | 1×10^6^ | Density gradient centrifugation | Vehicle |
| Altun 2012[10] | Turkey | RCT | SD rat | Male | 250-300g | / | 7/7 | Clip bilateral renal pedicles for 60 minutes | MSCs | Bone marrow of SD rats | Carotid artery | 1.5×10^6^ | Density gradient centrifugation | PBS |
| Cai 2014[11] | China | RCT | SD rat | Male | 250-300g | / | 12/12 | Clip bilateral renal pedicles for 45 minutes | MSCs | Bone marrow of SD rats | Carotid artery | 1×10^6^ | Adherent screening | PBS |
| Du 2012[12] | China | RCT | SD rat | Male | 200-220g | Adult | 16/16 | The left renal pedicle was clamped for 45 minutes, and the right nephrectomy was performed | MSCs | Human umbilical cord | Tail vein | 2×10^6^ | Adherent screening | Vehicle |
| Manna 2011[13] | Italy | RCT | SD rat | Male | 230-280g | / | 9/9 | Clip bilateral renal pedicles for 45 minutes | MSCs | Human fetal membranes | Renal cortex | 1×10^6^ | Tissue digestion | Normal saline |
| Tsuda 2014[14] | Japan | RCT | Lewis rat | Male | 230-250g | 10 weeks | 9/9 | The left renal pedicle was clamped for 45 minutes, and the right nephrectomy was performed | MSCs | Fetal membranes of SD rats | Tail vein | 0.5×10^6^ | Adherent screening | PBS |
| Sherif 2015[15] | Egypt | RCT | Wistar rat | Male | 200-250g | / | 10/10 | Cisplatin | MSCs | Bone marrow of Wistar rat | Tail vein | 2×10^6^ | Density gradient centrifugation | PBS |
| Karimi 2021[16] | Iran | RCT | SD rat | Male | 260-280g | / | 10/10 | Clip bilateral renal pedicles for 45 minutes | MSCs | Bone marrow of SD rats | Tail vein | 1.5×10^6^ | Adherent screening | Blank |
| Awadalla 2022[17] | Egypt | RCT | SD rat | Male | 200-250g | / | 30/30 | Cisplatin | MSCs | Adipose tissue around epididymis in SD rats | Tail vein | 1×10^6^ | Adherent screening | Normal saline |
| Zeinali 2021[18] | Iran | RCT | Wistar rat | Male | 150-200g | / | 12/12 | Cisplatin | MSCs | Human endometrium | Tail vein | 1×10^6^ | Density gradient centrifugation | PBS |
| Sheashaa 2016[19] | Egypt | RCT | SD rat | Male | 250-300g | / | 24/24 | The left renal pedicle was clamped for 45 minutes, and the right nephrectomy was performed | MSCs | Adipose tissue around epididymis in SD rats | Tail vein | 1×10^6^ | Flow cytometry separation | Normal saline |
| Xu 2020[20] | China | RCT | SD rat | Male | 250±10g | 8 weeks | 6/6 | Cisplatin | MSCs | Human umbilical cord | Femoral vein | 2×10^6^ | Flow cytometry separation | Normal saline |
| Havakhah 2018[21] | Iran | RCT | Wistar rat | Male | 250-300g | Adult | 5/5 | Clip bilateral renal pedicles for 40 minutes | MSCs | Bone marrow of Wistar rat | Renal cortex | 2×10^6^ | Adherent screening | DMEM |
| Changizi 2020[22] | Iran | RCT | Wistar rat | Male | 180-200g | / | 7/7 | Clip bilateral renal pedicles for 45 minutes | MSCs | Adipose tissue around epididymis in SD rats | Tail vein | 2×10^6^ | Adherent screening | PBS |
| Gao 2012[23] | China | RCT | SD rat | Male | 200g | / | 180 | Clip bilateral renal pedicles for 40 minutes | MSCs | Subcutaneous adipose tissue of SD rats | Renal cortex | 2×10^6^ | Flow cytometry separation | PBS |
| Zhuo 2013[24] | China | RCT | SD rat | Male | 250-300g | 8-10 weeks | 8/8 | The right renal pedicle was clamped for 60 minutes, and the left kidney was removed | MSCs | Bone marrow of SD rats | Renal artery | 1×10^6^ | Adherent screening | DMEM |
| Wang 2013[25] | China | RCT | Wistar rat | Male | 250-300g | Adult | 6/6 | Clip bilateral renal pedicles for 30 minutes | MSCs | Inguinal adipose tissue in Wistar rats | Penile veins | 2×10^6^ | Flow cytometry separation | PBS |
| Zhao 2014[26] | China | RCT | SD rat | Male | 200-300g | Adult | 10/10 | Clip bilateral renal pedicles for 60 minutes | MSCs | Bone marrow of SD rats | Renal cortex | 1×10^6^ | Density gradient centrifugation | Blank |
| Feng 2010[27] | Americaa | RCT | Fisher 344 rat | Male | 200-300g | Adult | 8/8 | Clip bilateral renal pedicles for 60 minutes | MSCs | Inguinal adipose tissue of Fisher 344 rats | Renal artery | 5×10^6^ | Tissue digestion | PBS |
| Cai 2014[28] | China | RCT | SD rat | Male | 250-300g | / | 6/6 | The left renal pedicle was clamped for 45 minutes, and the right nephrectomy was performed | MSCs | Bone marrow of SD rats | Renal artery | 1×10^6^ | Flow cytometry separation | Blank |
| Shih 2013[29] | China | RCT | SD rat | Male | 300-350g | 8 weeks | 5/5 | The left renal pedicle was clamped for 45 minutes, and the right nephrectomy was performed | MSCs | Inguinal adipose tissue in SD rats | Femoral vein、Renal artery | 0.5×10^6^ | Tissue digestion | PBS |
| Ashour 2016[30] | Egypt | RCT | SD rat | Male | / | / | 10/10 | Cisplatin | MSCs | Bone marrow of SD rats | Tail vein | 5×10^6^ | Density gradient centrifugation | PBS |
| Zhou 2020[31] | China | RCT | SD rat | Male | 200-250g | / | 10/10 | Clip bilateral renal pedicles for 45 minutes | MSCs | Bone marrow of SD rats | Tail vein | 1×10^6^ | Flow cytometry separation | DMEM |
| Zhang 2018[32] | China | RCT | SD rat | Female | 180-220g | Adult | 6/6 | Cisplatin | MSCs | Human umbilical cord | Tail vein | 1×10^6^ | Tissue digestion | PBS |
| Chen 2011[33] | China | RCT | SD rat | Female | 200-250g | Adult | 6/6 | Clip bilateral renal pedicles for 60 minutes | MSCs | Human umbilical cord | Carotid artery | 1×10^6^ | Tissue digestion | PBS |
| Chen 2011[34] | China | RCT | SD rat | Male | 275-300g | Adult | 8/8 | Clip bilateral renal pedicles for 60 minutes | MSCs | Adipose tissue around epididymis in SD rats | Penile veins | 1×10^6^ | Tissue digestion | Blank |
| Zhou 2017[35] | China | RCT | SD rat | Male | 220-250g | / | 8/8 | The left renal pedicle was clamped for 45 minutes, and the right nephrectomy was performed | MSCs | Human perirenal adipose tissue | Renal parenchyma | 2×10^6^ | Density gradient centrifugation | PBS |
| Huang 2019[36] | China | RCT | SD rat | Male | 220-250g | 9 weeks | 15/15 | Clip bilateral renal pedicles for 40 minutes | MSCs | Human perirenal adipose tissue | Renal cortex | 2×10^6^ | Tissue digestion | Normal saline |
| Guo 2018[37] | China | RCT | Wistar rat | Female | / | 6-8 weeks | 18/18 | Clip bilateral renal pedicles for 45 minutes | MSCs | Umbilical cord of pregnant rat | Tail vein | 1×10^6^ | Tissue digestion | Blank |
| Hussein 2016[38] | Egypt | RCT | SD rat | Male | 220-250g | 12-16 weeks | 18/18 | The left renal pedicle was clamped for 45 minutes, and the right nephrectomy was performed | MSCs | Adipose tissue adjacent to the scrotum in Wistar rats | Penile veins | 1×10^6^ | Density gradient centrifugation | PBS |
| Liu 2013[39] | China | RCT | SD rat | Male | 250-300g | / | 30/30 | Clip bilateral renal pedicles for 45 minutes | MSCs | Inguinal adipose tissue in SD rats | Renal artery | 0.5×10^6^ | Tissue digestion | PBS |
| Huang 2012[40] | China | RCT | SD rat | Male | 200g | / | 18/18 | Clip bilateral renal pedicles for 60 minutes | MSCs | Bone marrow of SD rats | Tail vein | 5×10^6^ | Flow cytometry separation | PBS |
| Liu 2008[41] | China | RCT | SD rat | Female | 200-250g | / | 30/30 | Gentamicin | MSCs | Bone marrow of SD rats | Tail vein | 5×10^6^ | Adherent screening | DMEM |
| Huang 2012[42] | China | RCT | SD rat | Male | 275-300g | Adult | 8/8 | Clip bilateral renal pedicles for 60 minutes | MSCs | Inguinal adipose tissue in SD rats | Tail vein | 1×10^6^ | Flow cytometry separation | PBS |
| Zhang 2021[43] | China | RCT | SD rat | Male | 350-370g | 11-12weeks | 8/8 | Clip bilateral renal pedicles for 40 minutes | MSCs | Inguinal adipose tissue in SD rats | Tail vein | 1×10^6^ | Flow cytometry separation | PBS |
| Xi 2018[44] | China | RCT | SD rat | Male | 200-250g | 6-8 weeks | 14/12 | Clip bilateral renal pedicles for 45 minutes | MSCs | Bone marrow of SD rats | Inferior vena cava | 1×10^7^ | Adherent screening | PBS |
| Zhu 2012[45] | China | RCT | SD rat | Male | 180-220g | 9 weeks | 15/15 | Clip bilateral renal pedicles for 45 minutes | MSCs | Bone marrow of SD rats | Tail vein | 1.5×10^7^ | Adherent screening | PBS |
| Ma 2017[46] | China | RCT | SD rat | Male | 180-220g | 8 weeks | 12/12 | Clip bilateral renal pedicles for 45 minutes | MSCs | Human umbilical cord | Tail vein | 2×10^6^ | Adherent screening | DMEM |
| Wang 2015[47] | China | RCT | SD rat | Male | 180-220g | / | 20/20 | Clip bilateral renal pedicles for 40 minutes | MSCs | Human umbilical cord | Tail vein | 1×10^7^ | Tissue digestion | DMEM |
| Li 2010[48] | China | RCT | SD rat | Male | 200-250g | / | 40/40 | Gentamicin | MSCs | Human umbilical cord | Tail vein | 1.5×10^7^ | Adherent screening | DMEM |
| Zhang 2015[49] | China | RCT | SD rat | Male | 200g | 12weeks | 10/10 | Clip bilateral renal pedicles for 45 minutes | MSCs | Human umbilical cord | Tail vein | 1×10^6^ | Adherent screening | Normal saline |
| Lei 2019[50] | China | RCT | SD rat | Male | 275-300g | 8-10 weeks | 15/15 | The left renal pedicle was clamped for 45 minutes, and the right nephrectomy was performed | MSCs | Inguinal adipose tissue in SD rats | Tail vein | 0.5×10^6^ | Adherent screening | Normal saline |

[1] J.H. Sun, G.J. Teng, Z.L. Ma, S.H. Ju, In vivo monitoring of magnetically labeled mesenchymal stem cells administered intravascularly in rat acute renal failure, Swiss Med Wkly 138(27-28) (2008) 404-12.

[2] G.H. Xiu, X. Zhou, X.L. Li, X.Z. Chen, B.Q. Li, X.L. Chen, H. Jin, X.H. Pan, J. Sun, B. Ling, Role of Bone Marrow Mesenchymal Stromal Cells in Attenuating Inflammatory Reaction in Lipopolysaccaride-induced Acute Kidney Injury of Rats Associated with TLR4-NF-kappaB Signaling Pathway Inhibition, Ann Clin Lab Sci 48(6) (2018) 743-750.

[3] J.B. Zhang, X.Q. Wang, G.L. Lu, H.S. Huang, S.Y. Xu, Adipose-derived mesenchymal stem cells therapy for acute kidney injury induced by ischemia-reperfusion in a rat model, Clin Exp Pharmacol Physiol 44(12) (2017) 1232-1240.

[4] Y.T. Chen, C.K. Sun, Y.C. Lin, L.T. Chang, Y.L. Chen, T.H. Tsai, S.Y. Chung, S. Chua, Y.H. Kao, C.H. Yen, P.L. Shao, K.C. Chang, S. Leu, H.K. Yip, Adipose-derived mesenchymal stem cell protects kidneys against ischemia-reperfusion injury through suppressing oxidative stress and inflammatory reaction, J Transl Med 9 (2011) 51.

[5] W. Zhang, L. Liu, Y. Huo, Y. Yang, Y. Wang, Hypoxia-pretreated human MSCs attenuate acute kidney injury through enhanced angiogenic and antioxidative capacities, Biomed Res Int 2014 (2014) 462472.

[6] X. Zhao, X. Qiu, Y. Zhang, S. Zhang, X. Gu, H. Guo, Three-Dimensional Aggregates Enhance the Therapeutic Effects of Adipose Mesenchymal Stem Cells for Ischemia-Reperfusion Induced Kidney Injury in Rats, Stem Cells Int 2016 (2016) 9062638.

[7] L. Hafazeh, S. Changizi-Ashtiyani, F. Ghasemi, H. Najafi, S. Babaei, F. Haghverdi, Stem Cell Therapy Ameliorates Ischemia-reperfusion Induced Kidney Injury After 24 Hours Reperfusion, Iran J Kidney Dis 13(6) (2019) 380-388.

[8] P. Erpicum, P. Rowart, L. Poma, J.M. Krzesinski, O. Detry, F. Jouret, Administration of mesenchymal stromal cells before renal ischemia/reperfusion attenuates kidney injury and may modulate renal lipid metabolism in rats, Sci Rep 7(1) (2017) 8687.

[9] W. Zhuo, L. Liao, T. Xu, W. Wu, S. Yang, J. Tan, Mesenchymal stem cells ameliorate ischemia-reperfusion-induced renal dysfunction by improving the antioxidant/oxidant balance in the ischemic kidney, Urol Int 86(2) (2011) 191-6.

[10] B. Altun, R. Yilmaz, T. Aki, H. Akoglu, D. Zeybek, S. Piskinpasa, D. Uckan, N. Purali, P. Korkusuz, C. Turgan, Use of mesenchymal stem cells and darbepoetin improve ischemia-induced acute kidney injury outcomes, Am J Nephrol 35(6) (2012) 531-9.

[11] J. Cai, X. Yu, B. Zhang, H. Zhang, Y. Fang, S. Liu, T. Liu, X. Ding, Atorvastatin improves survival of implanted stem cells in a rat model of renal ischemia-reperfusion injury, Am J Nephrol 39(6) (2014) 466-75.

[12] T. Du, J. Cheng, L. Zhong, X.F. Zhao, J. Zhu, Y.J. Zhu, G.H. Liu, The alleviation of acute and chronic kidney injury by human Wharton's jelly-derived mesenchymal stromal cells triggered by ischemia-reperfusion injury via an endocrine mechanism, Cytotherapy 14(10) (2012) 1215-27.

[13] G. La Manna, F. Bianchi, M. Cappuccilli, G. Cenacchi, L. Tarantino, G. Pasquinelli, S. Valente, E. Della Bella, S. Cantoni, C. Claudia, F. Neri, M. Tsivian, B. Nardo, C. Ventura, S. Stefoni, Mesenchymal stem cells in renal function recovery after acute kidney injury: use of a differentiating agent in a rat model, Cell Transplant 20(8) (2011) 1193-208.

[14] H. Tsuda, K. Yamahara, K. Otani, M. Okumi, K. Yazawa, J.Y. Kaimori, A. Taguchi, K. Kangawa, T. Ikeda, S. Takahara, Y. Isaka, Transplantation of allogenic fetal membrane-derived mesenchymal stem cells protects against ischemia/reperfusion-induced acute kidney injury, Cell Transplant 23(7) (2014) 889-99.

[15] I.O. Sherif, L.A. Al-Mutabagani, A.M. Alnakhli, M.A. Sobh, H.E. Mohammed, Renoprotective effects of angiotensin receptor blocker and stem cells in acute kidney injury: Involvement of inflammatory and apoptotic markers, Exp Biol Med (Maywood) 240(12) (2015) 1572-9.

[16] Z. Karimi, S. Janfeshan, E. Kargar Abarghouei, S.S. Hashemi, Therapeutic effects of bone marrow mesenchymal stem cells via modulation of TLR2 and TLR4 on renal ischemia-reperfusion injury in male Sprague-Dawley rats, Bioimpacts 11(3) (2021) 219-226.

[17] A. Awadalla, A.M. Hussein, Y.M. El-Far, F.F. El-Senduny, N. Barakat, E.T. Hamam, H.M. Abdeen, M. El-Sherbiny, M.S. Serria, A.A. Sarhan, A.M. Sena, A.A. Shokeir, Rapamycin Improves Adipose-Derived Mesenchymal Stem Cells (ADMSCs) Renoprotective Effect against Cisplatin-Induced Acute Nephrotoxicity in Rats by Inhibiting the mTOR/AKT Signaling Pathway, Biomedicines 10(6) (2022).

[18] H. Zeinali, M. Azarnia, P. Keyhanvar, R. Moghadasali, S. Ebrahimi-Barough, Human Endometrial Stromal/Stem Cells Inhibit Apoptosis in Cisplatin-Induced Acute Kidney Injury in Male Wistar Rats, Cell J 23(5) (2021) 568-575.

[19] H. Sheashaa, A. Lotfy, F. Elhusseini, A.A. Aziz, A. Baiomy, S. Awad, A. Alsayed, A.H. El-Gilany, M.A. Saad, K. Mahmoud, F. Zahran, D.A. Salem, A. Sarhan, H.A. Ghaffar, M. Sobh, Protective effect of adipose-derived mesenchymal stem cells against acute kidney injury induced by ischemia-reperfusion in Sprague-Dawley rats, Exp Ther Med 11(5) (2016) 1573-1580.

[20] Q. Xu, P. Yan, X.J. Duan, X. Wu, X.J. Chen, M. Luo, J.C. Peng, L.X. Feng, J. Liu, H.L. Zhong, W. Cheng, Q.Y. Zou, S.B. Duan, Human umbilical cord-derived mesenchymal stem cells and human cord blood mononuclear cells protect against cisplatin-induced acute kidney injury in rat models, Exp Ther Med 20(6) (2020) 145.

[21] S. Havakhah, M. Sankian, G.H. Kazemzadeh, K. Sadri, H.R. Bidkhori, H. Naderi-Meshkin, A. Ebrahimzadeh Bideskan, S. Niazmand, A.R. Bahrami, A. Khajavi Rad, In vivo effects of allogeneic mesenchymal stem cells in a rat model of acute ischemic kidney injury, Iran J Basic Med Sci 21(8) (2018) 824-831.

[22] S. Changizi-Ashtiyani, L. Hafazeh, F. Ghasemi, H. Najafi, S. Babaei, F. JalallyMashayekhi, S.J. Hoseini, B. Bastani, The effect of adipose-derived mesenchymal stem cells on renal function and histopathology in a rat model of ischemia-reperfusion induced acute kidney injury, Iran J Basic Med Sci 23(8) (2020) 999-1006.

[23] J. Gao, R. Liu, J. Wu, Z. Liu, J. Li, J. Zhou, T. Hao, Y. Wang, Z. Du, C. Duan, C. Wang, The use of chitosan based hydrogel for enhancing the therapeutic benefits of adipose-derived MSCs for acute kidney injury, Biomaterials 33(14) (2012) 3673-81.

[24] W. Zhuo, L. Liao, Y. Fu, T. Xu, W. Wu, S. Yang, J. Tan, Efficiency of endovenous versus arterial administration of mesenchymal stem cells for ischemia-reperfusion-induced renal dysfunction in rats, Transplant Proc 45(2) (2013) 503-10.

[25] Y.L. Wang, G. Li, X.F. Zou, X.B. Chen, T. Liu, Z.Y. Shen, Effect of autologous adipose-derived stem cells in renal cold ischemia and reperfusion injury, Transplant Proc 45(9) (2013) 3198-202.

[26] J.J. Zhao, J.L. Liu, L. Liu, H.Y. Jia, Protection of mesenchymal stem cells on acute kidney injury, Mol Med Rep 9(1) (2014) 91-6.

[27] Z. Feng, J. Ting, Z. Alfonso, B.M. Strem, J.K. Fraser, J. Rutenberg, H.C. Kuo, K. Pinkernell, Fresh and cryopreserved, uncultured adipose tissue-derived stem and regenerative cells ameliorate ischemia-reperfusion-induced acute kidney injury, Nephrol Dial Transplant 25(12) (2010) 3874-84.

[28] J. Cai, X. Yu, R. Xu, Y. Fang, X. Qian, S. Liu, J. Teng, X. Ding, Maximum efficacy of mesenchymal stem cells in rat model of renal ischemia-reperfusion injury: renal artery administration with optimal numbers, PLoS One 9(3) (2014) e92347.

[29] Y.C. Shih, P.Y. Lee, H. Cheng, C.H. Tsai, H. Ma, D.C. Tarng, Adipose-derived stem cells exhibit antioxidative and antiapoptotic properties to rescue ischemic acute kidney injury in rats, Plast Reconstr Surg 132(6) (2013) 940e-951e.

[30] R.H. Ashour, M.A. Saad, M.A. Sobh, F. Al-Husseiny, M. Abouelkheir, A. Awad, D. Elghannam, H. Abdel-Ghaffar, M. Sobh, Comparative study of allogenic and xenogeneic mesenchymal stem cells on cisplatin-induced acute kidney injury in Sprague-Dawley rats, Stem Cell Res Ther 7(1) (2016) 126.

[31] S. Zhou, Y.M. Qiao, Y.G. Liu, D. Liu, J.M. Hu, J. Liao, M. Li, Y. Guo, L.P. Fan, L.Y. Li, M. Zhao, Bone marrow derived mesenchymal stem cells pretreated with erythropoietin accelerate the repair of acute kidney injury, Cell Biosci 10(1) (2020) 130.

[32] R. Zhang, L. Yin, B. Zhang, H. Shi, Y. Sun, C. Ji, J. Chen, P. Wu, L. Zhang, W. Xu, H. Qian, Resveratrol improves human umbilical cord-derived mesenchymal stem cells repair for cisplatin-induced acute kidney injury, Cell Death Dis 9(10) (2018) 965.

[33] Y. Chen, H. Qian, W. Zhu, X. Zhang, Y. Yan, S. Ye, X. Peng, W. Li, W. Xu, Hepatocyte growth factor modification promotes the amelioration effects of human umbilical cord mesenchymal stem cells on rat acute kidney injury, Stem Cells Dev 20(1) (2011) 103-13.

[34] Y.T. Chen, C.C. Yang, Y.Y. Zhen, C.G. Wallace, J.L. Yang, C.K. Sun, T.H. Tsai, J.J. Sheu, S. Chua, C.L. Chang, C.L. Cho, S. Leu, H.K. Yip, Cyclosporine-assisted adipose-derived mesenchymal stem cell therapy to mitigate acute kidney ischemia-reperfusion injury, Stem Cell Res Ther 4(3) (2013) 62.

[35] L. Zhou, Q. Song, J. Shen, L. Xu, Z. Xu, R. Wu, Y. Ge, J. Zhu, J. Wu, Q. Dou, R. Jia, Comparison of human adipose stromal vascular fraction and adipose-derived mesenchymal stem cells for the attenuation of acute renal ischemia/reperfusion injury, Sci Rep 7 (2017) 44058.

[36] X. Huang, H. Wang, Y. Xu, Induced Pluripotent Stem Cells (iPSC)-derived Mesenchymal Stem Cells (MSCs) Showed Comparable Effects in Repair of Acute Kidney Injury as Compared to Adult MSCs, Urol J 17(2) (2020) 204-209.

[37] Q. Guo, J. Wang, Effect of combination of vitamin E and umbilical cord-derived mesenchymal stem cells on inflammation in mice with acute kidney injury, Immunopharmacol Immunotoxicol 40(2) (2018) 168-172.

[38] A.M. Hussein, N. Barakat, A. Awadalla, M.M. Gabr, S. Khater, A.M. Harraz, A.A. Shokeir, Modulation of renal ischemia/reperfusion in rats by a combination of ischemic preconditioning and adipose-derived mesenchymal stem cells (ADMSCs), Can J Physiol Pharmacol 94(9) (2016) 936-46.

[39] S.P. Liu, X.F. Yu, Y.H. Zhong, Y. Fang, H. Liu, R. Xu, J. Guo, J.R. Cai, T.Q. Liu, T. Xie, X.Q. Ding, Transplantation of adipose-derived stem cells via renal artery protects against acute ischemic kidney injury, Chin J Nephrol 29(10) (2013) 768-774.

[40] F.H. Huang, X.M. Zheng, W.Q. Yao, Repair of acute ischemia-reperfusion renal injury by bone marrow mesenchymal stem cells transplantation in rats, Chin J Exp Surg 29(11) (2012) 2246-2248.

[41] H.B. Liu, F. Zhao, W. Chen, H.M. Wang, P. Zhang, G.S. Xu, X.W. Liu, Y. Yu, G.L. Chen, F. Zhang, Protective effects of bone marrow mesenchymal stem cells in rat with gentamicin induced acute renal failure, Chin J Emerg Med 17(2) 2008 162-166.

[42] F.H. Huang, X.M. Zheng, W.Q. Yao, Protective effect of autologous adipose-derived mesenchymal stem cells on renal ischemia reperfusion injury in rats, Chin J Exp Surg 29(12) (2012) 2419-2421.

[43] J.B. Zhang, C.Y. Lin, L. Wan, S.Y. Xu, Protective effect of adipose ⁃ derived mesenchymal stem cells combined with dexmedetomidine for acute ischemia ⁃ reperfusion injury in rats, The Journal of Practical Medicine 37(23) 2971-2976.

[44] X.Q. Xi, H.L. Hu, C. Zou, Z.Q. Fang, R.R. Kuang, Z.F. Ye, Y.W. Huang, Bone marrow mesenchymal stem cells stably expressing NICD which is controlled by Tet-On system in the treatment of renal ischemia-reperfusion injury, Chinese Journal of Tissue Engineering Research 22(25) 2018 4035-4040.

[45] F.R. Zhu, J.D. Jiao, S.Y. Liu, C.M. L, Bone marrow mesenchymal stem cell transplantation for acute kidney injury induced by excessive exercise, CJITWN 13(9) 805-806.

[46] H.L. Ma, Y. Xu, R.R. Zhang, S.Y. Zhang, X.Z. Zhang, The therapeutic effect of mesenchymal stem cells on endotoxin - induced acute kidney injury in rats, Journal of Jinan University (Natural Science ＆ Medicine Edition), 38(3) 2017 253-258.

[47] T.S. Wang, J.J. Zhang, Human telomerase reverse transcriptase gene-modified umbilical cord mesenchymal stem cell transplantation for acute kidney injury, Chinese Journal of Tissue Engineering Research 19(23) 2015 3686-3691.

[48] F. Li, Z.J. Dang, X. Hu, Y.N. Zhou, Distribution of the human umbilical cord mesenchymal stem cells after transplantation in rats with acute tubular necrosis, J of Pub Health and Prev Med 201021(5) 5-9.

[49] B. Zhang, W.Q. Li, Homing of human umbilical cord-derived mesenchymal stem cells to the injured kidney and their protective effects, Chinese Journal of Tissue Engineering Research 2015 19(45) 7304-7308.

[50] Y. Lei, R.A. Liu, F. Zeng, Underlying mechanism by which adipose-derived mesenchymal stem cell transplantation alleviates renal ischemia-reperfusion injury in rats, Chinese Journal of Tissue Engineering Research 2019 23(5) 749-755.
